# The fall descriptions and health characteristics of older adults with hip fracture: a mixed methods study

**DOI:** 10.1186/s12877-015-0036-x

**Published:** 2015-04-08

**Authors:** Breiffni Leavy, Liisa Byberg, Karl Michaëlsson, Håkan Melhus, Anna Cristina Åberg

**Affiliations:** 1 Department of Surgical Sciences, Orthopedics, Uppsala University, Uppsala, Sweden; 2 Department of Medical Sciences, Osteoporosis and Clinical Pharmacogenetics, Uppsala University, Uppsala, Sweden; 3 Department of Public Health and Caring Sciences, Geriatrics, Uppsala University, Uppsala, Sweden; 4 School of Education, Health and Society, Dalarna University, Falun, Sweden

**Keywords:** Hip fracture, Fall circumstances, Health and functional characteristics

## Abstract

**Background:**

In light of the multifactorial etiology of fall-related hip fracture, knowledge of fall circumstances may be especially valuable when placed in the context of the health of the person who falls. We aimed to investigate the circumstances surrounding fall-related hip fractures and to describe fall circumstances in relation to participants’ health and functional characteristics.

**Methods:**

The fall circumstances of 125 individuals (age ≥ 50 years) with hip fracture were investigated using semi-structured interviews. Data concerning participants’ health (comorbidities and medications) and function (self-reported performance of mobility, balance, personal activities of daily living and physical activity, previous falls and hand grip strength) were collected via medical records, questionnaires and dynamometry. Using a mixed methods design, both data sets were analysed separately and then merged in order to provide a comprehensive description of fall events and identify eventual patterns in the data.

**Results:**

Fall circumstances were described as i) Activity at the time of the fall: Positional change (n = 24, 19%); Standing (n = 16, 13%); Walking (n = 71, 57%); Balance challenging (n = 14, 11%) and ii) Nature of the fall: Environmental (n = 32, 26%); Physiological (n = 35, 28%); Activity-related indoor (n = 8, 6%) and outdoor (n = 8, 6%); Trips and slips on snow (n = 20, 16%) and in snow-free conditions (n = 12, 10%) and Unknown (n = 10, 8%)*.* We observed the following patterns regarding fall circumstances and participants’ health: those who fell i) during positional change had the poorest functional status; ii) due to environmental reasons (indoors) had moderate physical function, but high levels of comorbidity and fall risk increasing medications; iii) in snow-free environments (outdoors) appeared to have a poorer health and functional status than other outdoor groups.

**Conclusions:**

Our findings indicate that patterns exist in relation to the falls circumstances and health characteristics of people with hip fracture which build upon that previously reported. These patterns, when verified, can provide useful information as to the ways in which fall prevention strategies can be tailored to individuals of varying levels of health and function who are at risk for falls and hip fracture.

**Electronic supplementary material:**

The online version of this article (doi:10.1186/s12877-015-0036-x) contains supplementary material, which is available to authorized users.

## Background

Hip fractures are among the most sinister outcomes of falls in elderly populations due to detrimental effects on functional capacity [1] independence [2] and mortality [3]. The vast majority of hip fractures are preceded by a fall, so predisposing risk factors for falls and hip fractures are in many cases similar [4-8]. While predisposing risk factors such as high age and comorbidities indicate susceptibility to hip fracture, they do not explain the ways in which the falls which precede hip fractures occur. To understand the circumstances surrounding fall-related hip fractures the precipitating factors for these falls need to be examined and evidence for these is sparse in the literature.

Previous studies which provide the most detailed accounts of fall circumstances have been based in residential care settings [9-13]. Based on a research teams analysis of video-captured falls, incorrect shifting of body weight, followed by trips and stumbles were reported as the primary causes of imbalance [10]. When resident clinicians have, on the other hand, analysed falls not captured on video, symptoms of acute disease have been cited as the primary precipitating fall risk factors [11]. Although third party observations of falls in residential care settings provide valuable insights into fall mechanisms, they do not account for subjectively experienced factors such as dizziness or pain, or provide contextual health details of the person who fell [9,13]. Furthermore, these studies are not generalizable to all patients with hip fracture since the majority of hip fractures occur unobserved in community settings [14]. Findings from interview studies which have explored older people’s perspectives on falling, on the other hand, describe a diversity of fall perceptions involving both individual [15,16] and environmental or accidental precipitants [17,18]. Explorative studies however, rarely place these perceptions in the context of the health of the faller [17,19-21], which may explain these reported diversities in fall perceptions [22].

Characteristics of poor health and function are known to predispose to falls and hip fractures. Physical function is an indicator of health among older people [23] and is commonly measured using self-reported performance of mobility, personal activities of daily living (P-ADL), balance, and physical activity (PA) [24-26]. A non-linear relationship is reported between gait speed and falls, with those at lower and higher levels at greatest risk [27], a relationship possibly mediated by exposure to environmental hazards [28]. Impaired balance [29,30] and muscle weakness, particular of the lower limb [31], are also associated with increased risk for falls and hip fractures. Additionally, impairments in physical function may be the manifestation of underlying chronic disease, or the side effects of drugs which treat disease, thus comorbidity and medication use are important factors for consideration in fall investigations [32,33]. Hip fracture is, in almost all instances, preceded by a fall and treated in hospital. By approaching all patients with incident hip fracture, it is therefore possible to identify a population based sample of persons who have experienced a fall.

Fall circumstances and the predisposing health characteristics of those who fall have, to date, mostly been investigated in separate studies. Placing fall descriptions within the health context of the person who falls may help identify important patterns, which can inform preventive efforts. The main purpose of this study was to investigate the circumstances surrounding fall-related hip fractures and to describe these circumstances in relation to participants’ health and functional characteristics. More specifically, we aimed to explore the fall descriptions of patients, without cognitive impairment, who were admitted to a Swedish hospital with hip fracture during a 10-month period. Based on qualitative content analysis, categories were created and we then aimed to explore whether health and functional characteristics varied across these categories and whether there were identifiable patterns in the data.

## Methods

### Design

This descriptive study was explorative in nature and used a concurrent triangulation mixed methods design [34]. With this design, qualitative and quantitative data are collected concurrently, analysed separately and then merged. The two forms of data were merged in the results section using a joint display of the data [35] and then interpreted in the discussion section. The rationale for this approach is that of complementarity whereby fall descriptions will explain one aspect of fall circumstances and quantitative health data are collected to elaborate and enhance these descriptions [36].

### Participants

All patients aged 50 years and over, with radiographically confirmed hip fracture (S72.0–S72.2 according to International Classification of Diseases (ICD-10)), admitted to a Swedish hospital during the period August 2009 − June 2010, were considered for inclusion in the study. The hospital is responsible for all acute care and surgery in the catchment area of Uppsala County, which enabled us to identify all hip fractures in this population during the study period. Participants were enrolled consecutively during hospital stay, if they fitted the following inclusion criteria; memory of the fall and verbal ability to recount its details and a Mini-mental State Examination (MMSE) score ≥24 points. Those with reduced consciousness, post-operative confusion or medical instability were not considered for participation, in this otherwise population-based setting. The interviews were performed during hospital stay within a one week period of when the fall event had occurred.

Of the 350 patients with hip fracture approached during the 10-month period, 192 people were unfit for interview due to; diagnosed dementia, communication difficulties or postoperative complications such as reduced consciousness, confusion, or medical instability. A further 29 patients were excluded following MMSE (score <24 points), leaving 129 participants eligible for interview. Following initial interviews analysis four participants were excluded due to fall recall uncertainty. Pre-fracture characteristics of the 125 men and women aged 55–96 years included in the present study are presented in Table 1.

**Table 1:** **Characteristics of 125 interviewed participants**

	Male	Female	Total
	36 (29.7)	89 (70.3)	n = 125
**Demographics**			
Age, years (mean(SD))	77.6 (9.1)	79.7 (9.5)	79.1 (9.4)
Residential status			
Community-dwelling	34 (94.4)	83 (93.2)	116 (92.8)
Serviced apartment	1 (2.8)	2 (2.3)	3 (2.4)
Residential care	1 (2.8)	4 (4.5)	5 (4.0)
**BMI (n = 109)**			
Underweight	2 (6.3)	11 (14.3)	13 (11.9)
Normal weight	16 (50.0)	44 (57.1)	60 (55.1)
Overweight/Obese	14 (43.7)	22 (28.6)	36 (33.0)
**Mobility** ^**1**^			
Low^2^	10 (27.8)	26 (29.2)	36 (28.8)
Moderate^3^	5 (13.9)	26 (29.2)	31 (24.8)
High^4^	21 (58.3)	37 (41.6)	58 (46.4)
**P-ADL Participation** ^**1**^			
Dependent in ≥1 activity of personal care	5 (13.9)	8 (8.9)	13 (10.4)
**Balance** ^**1**^			
Self-rated balance (bad), (n = 114)	20 (62.5)	39 (47.6)	59 (51.75)
Fear of falling (yes), (n = 122)	9 (26.5)	27 (30.7)	36 (29.5)
**Previous falls**			
**≥**1 fall previous year, (n = 122)	23 (63.9)	37 (43.0)	60 (49.2)
**Physical activity**^**1**^ (n = 118)			
Sedentary	8 (22.2)	14 (15.7)	22 (17.6)
Light exercise	26 (72.2)	67 (75.3)	93 (74.4)
Hard physical training	1 (2.8)	2 (2.3)	3 (2.4)
**Grip strength** (n = 107)			
Normal (≥10th percentile)	17 (51.5)	43 (58.1)	60 (56.1)
Low (5th-10th percentile)	7 (21.2)	16 (21.6)	23 (21.5)
Abnormally low (<5th percentile)	9 (27.3)	15 (20.3)	24 (22.4)
**Number of chronic diseases** ^**5**^			
0	16 (44.4)	51 (57.3)	67 (53.6)
1	13 (33.3)	29 (32.6)	41 (32.8)
≥2	8 (22.2)	9 (10.1)	17 (13.6)
**Fall-risk-increasing drugs (FRIDs)**			
0 FRIDs	6 (16.7)	18 (20.2)	24 (19.2)
Cardiovascular (Cvd) FRIDs	13 (36.1)	35 (39.3)	48 (38.4)
Psychotropic (Psy) FRIDs	5 (13.9)	8 (8.9)	13 (10.4)
Concomitant Cvd & PsyFRIDs	12 (33.3)	28 (31.5)	40 (32.0)

Units are expressed as number (percentage) unless otherwise stated.

^1^Reported performance of pre-fracture status. ^2^Required a walking aid indoors. ^3^Required a walking aid outdoors. ^4^No walking aid required. ^5^According to Charlson’s unweighted comorbidity index.

Participants received written information about study details and were given time to read this information before choosing whether to participate or not. All participants gave their verbal and written consent for inclusion in the study. The study was approved by the Regional Ethics Committee in Uppsala, Sweden.

### Data collection

#### Fall circumstances

Qualitative interviews explored circumstances of the first two phases of falling, as outlined by Noury et al. [37] firstly the ‘pre-fall phase’ involves the last activity carried out before falling; secondly, the ‘critical phase’, involves the sudden movement of the body towards the ground, until contact is made with the ground or an obstacle. The final two of Noury et al.’s falling phases (the post-fall and recovery phase) were not in focus in the study. During semi-structured interviews, performed by the same investigator, participants were encouraged to speak freely while describing fall events. The interview opened with the question ‘Can you describe for me what happened when you fell and broke your hip?’ Follow-up questions also aimed to investigate the eventual influence of other specific environmental, individual or situational factors which may have played a role in the fall occurrence. Interviews varied in length from 8 to 25 minutes and were recorded and transcribed verbatim. Each interview was followed by an interview-administered questionnaire containing closed-answer questions concerning more specific details of the fall, e.g. time of the fall and use of mobility aids.

#### Health-related data

Data concerning pre-fracture functional status were collected by interview-administered questionnaire. Questions related to prior performance of P-ADL, mobility, balance, previous falls and PA participation and were categorized as described below. Hand grip strength was tested, by the same investigator, using the ‘Baseline’ hydraulic hand dynamometer (Fabrication Enterprises Inc.) with the elbow flexed and supported at 90 degrees and the bed backrest elevated to achieve a high-lying position. Participants performed three maximal grip contractions of the dominant hand and the amount of time to maintain the grip was not specified. The mean value of the 3 automatically recorded contractions (measured in kg force) was calculated and is considered a valid and reliable method of measuring muscle strength [38], in turn an indicator of functional capacity [39]. Data concerning pre-fracture comorbidity and medication were retrieved from medical records. Comorbidity was categorized as 0, 1 and ≥ 2 chronic diseases, based on Charlson’s unweighted comorbidity score, calculated from ICD-10 diagnoses [40,41]. Charlson’s comorbidity index predicts the 10 year mortality for a person who may have a range of comorbid conditions. Fall-risk-increasing drugs (FRIDs), i.e. drugs with the strongest evidence for increasing fall risk such as, for example, benzodiazepines, antidepressants or anticonvulsants [42,43], were categorized as: no FRIDs, psychotropic FRIDs (PsyFRID; Anatomical Therapeutic Chemical (ATC) classification system codes N02A, N03A, N04A-B, N05A-C, N06A), cardiovascular FRIDs (CvdFRID; ATC codes C01A, C01BA, C01D, C02, C03, C07-C09, G04CA) and concomitant use of PsyFRID and CvdFRID.

### Data analysis

#### Analysis of fall circumstances from interviews

All interviews were recorded and transcribed and then systematically analysed using qualitative content analysis, which is a replicable and valid method for making specific inferences from text [44]. The analysis focused on the manifest content of the data with the aim to produce descriptive categories, a process which involves interpretation at lower levels of abstraction [45]. The analysis was initiated by repeated reading of each interview to get a sense of the entirety [46]. Meaning units of analysis were then identified and constituted sentences in the text describing i) physical activities in the pre-fall phase and ii) circumstances and precipitating factors during the critical phase. These text units were then condensed and grouped on the basis of similarity to form sub-categories and then categories for both ‘Activity at the time of the fall (pre-fall phase)’ and ‘Nature of the fall (critical phase)’. Categories were labelled to reflect participant descriptions and in consideration of the literature, so as to enable a comparison of findings. To ensure validity, this manifest analysis was constantly checked and compared with the original interview data. The first author was responsible for the primary coding of all interviews and to ensure trustworthiness of the analysis, emergent categories were adjusted and refined during repeated peer-debriefing sessions between the first and last authors [47]. In these sessions, the last author acted as a peer to the first author through a process of review and discussion of the credibility of categories in the data. Interview analysis preceded and was performed independently of the analysis of additional fall details, such as time of fall and use of a walking aid, which were collected by questionnaire and summarized by means of percentage, frequency.

#### Analysis of health data from questionnaires, medical records and dynamometry

Pre-fracture mobility was divided into the categories ‘Low’, ‘Moderate’ or ‘High’ which were defined as follows: Low (required a walking aid indoors); Moderate (required a walking aid outdoors only); High (no walking aid required). P-ADL participation was categorized as independent/dependent in ≥ 1 activity of personal care, whereby for example participants who required assistance/supervision during bathing were considered dependent in one activity. With regards to PA participation, participants answering ‘hardly any exercise’ to the question ‘How much exercise did you engage in prior to the fracture?’ were classified as ‘Sedentary’, with other divisions including ‘Light exercise’ (Lighter exercise such as regular walks or gardening) or ‘Hard physical exercise’ (Do you engage in hard physical training or sport?). This question was based on a questionnaire, created in collaboration with the Swedish national institute of health which measures lifetime physical activity [48], and has been used in previous cohort studies of older Swedish populations [49,50]. Absolute grip strength values were compared with published normative grip strength data [51], and stratified according to age, gender and height. ‘Low’ grip strength incorporated values between the 10th and the 5th percentile of normative data and ‘Abnormally low’ grip strength as those under the 5th percentile of normative data. In this calculation, 7 individuals (1 man and 6 women) lacked height data so for these we used the median height in the sex-specific age group in our sample (women > =65y: 164 cm; men > =65y: 175 cm). This imputation was only used for the definition of low grip strength.

#### Merging of the fall circumstance and health data

Following separate analysis of the interview and health-related data, both data sets were interrelated. To facilitate the integration of the data sets, qualitative fall circumstance categories were treated as categorical variables. We organized the data in relation to the fall circumstance categories which were viewed in relation to variables of health and function using cross tabulation and descriptive statistics. This triangulation of data occurred during both the analysis and interpretation phases with the purpose of interrelating and identifying patterns in the data [52].

### Statistical analysis

Descriptive statistics are presented as mean (SD) for continuous variables and as number (proportion) for categorical variables. The descriptive tables directly display relations among the study categories [53] and numerical comparisons are presented. In addition, statistical comparisons between groups of fall circumstances were performed by using analysis of variance for age and Fisher’s exact test for categorical variables. Data management and statistical analyses were performed using Stata version 12 (StataCorp, College Station, TX, USA).

## Results

### Fall circumstances

#### Activity at the time of the fall (pre-fall phase)

Participant descriptions of activities during the pre-fall phase were grouped into the following four main categories (incorporating eight subcategories of locomotor tasks): *Positional change* (Sit-to-stand (STS) and Sit-to-walk (STW), 24 (19%); *Standing* (Standing still and Standing while bending/reaching), 16 (13%); *Walking* (Forward walking and Turning while walking), 71 (57%) and *Balance challenging* (Walking on stairs and ‘Hazardous’), 14 (11%). The subcategory ‘Hazardous’ incorporated falls during complex activities, such as falls from: bikes, stools, ladders or whilst running or sledging in slippery conditions. Approximately half of those requiring a walking aid before the fracture reported using the aid during the fall, with men reporting higher usage (7/10) than women (12/26).

#### Nature of the fall (critical phase)

During the analysis of described precipitating fall factors, indoor and outdoor falls were analysed separately (See textbox outlining citations from the interviews depicting categories for Nature of the fall). Indoor falls (n = 83, 66%) were grouped into three main categories; *Environmental (*n = 32, 26%) incorporated falls described as primarily precipitated by environmental conditions/objects and included the subcategories: trips over; mats (n = 5), thresholds (n = 3), and household objects (n = 10), slips on wet surfaces (n = 8) and inadequate footwear, (n = 7); *Physiological* (n = 35, 28%) incorporated falls described as precipitated by physiological factors, with no environmental components and included the subcategories: self-induced disequilibrium − a symptomless loss of balance during normal body movements (n = 14); falls preceded by physical symptoms such as dizziness or faintness (n = 13) and reduced function/pain of the lower limb (n = 6) and; *Activity-related indoor* (n = 8, 6%) incorporated falls precipitated by the complex nature of the activities engaged in, such as standing on one leg or on kitchen stools.

No outdoor falls were described as involving physiological precipitants and these falls (n = 42, 34%) were therefore divided into the categories; *Trips & slips, snow* (n = 20, 16%) slipping and tripping on snowy or icy surfaces and *Trips & slips, no snow* (n = 12, 10%), slipping and tripping on snow and ice-free surfaces; *Activity-related outdoor* (n = 8, 6%), falls during high speeds (n = 2) or from ladders (n = 3) or bikes (n = 3).

The category *Unknown* (n = 10, 8%) incorporated falls of unexplained nature occurring both indoors (8/10) and outdoors (2/10).

### Fall circumstances in relation to age and gender

Although men (especially aged 80+ years) were overrepresented among outdoor fractures (36% men vs 19% women), activity at the time of the fracture did not vary according to gender. When comparing younger (50–80 years) and older (80+ years) participants, younger people fractured more frequently during *balance challenging* activities (9/63 (14%) vs 5/62 (8%)), and older participants were somewhat more represented among those fracturing during *positional change (*16% vs 23%*)*. In relation to nature of the fall, women alone described their indoor falls as *activity-related* and younger participants more commonly fractured outdoors in snowy conditions (25% vs 6%).

### Patterns between fall circumstances and health characteristics

**Figure 1:**
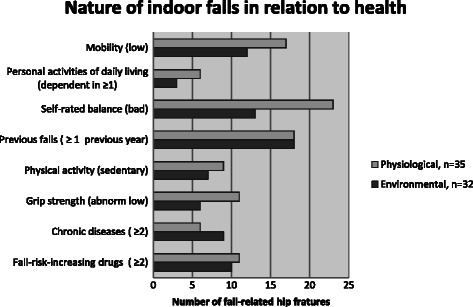
**Nature of the fall in relation to health characteristics for the two major indoor categories environmental and physiological.** Legend: P values for differences between categories were calculated using Fisher’s exact test. Mobility (low) p = 0.461, personal activities of daily living (dependent in ≥1) p = 0.477, self-rated balance (bad) p = 0.075, previous falls (≥1 previous year) p = 1.0, physical activity (sedentary) p = 0.774, grip strength (abnormally low) p = 0.380, chronic diseases (≥2) p = 0.381, fall-risk-increasing drugs (≥2) p = 1.0.

Using a mixed methods approach the fall circumstance categories ‘*Activity at the time of the fall*’ and ‘*Nature of the fall*’ were interrelated with participants’ health characteristics using descriptive statistics (Table 2, Figure 1 & Additional file 1: Table S1). Three main patterns were identified and will be discussed below.

**Table 2:** **Activity at the time of the fall in relation to health characteristics of 125 interviewed participants**

Health characteristic	Positional change	Standing	Walking	Balance challenging	Total	P for difference between groups
	n = 24	n = 16	n = 71	n = 14	n = 125	
Age, years (mean(SD))	81.4 (10.3)	78.7 (9.3)	79.4 (8.7)	76.7 (9.1)	79.1(9.4)	0.894
Gender (female)	16 (66.7)	11 (68.7)	52 (73.3)	10 (71.4)	89 (71.2)	0.919
**BMI (n = 109)**						0.466
Underweight	3 (16.7)	1 (7.1)	8 (12.7)	1 (7.1)	13 (11.9)	
Normal weight	7 (38.9)	11 (78.6)	33 (52.4)	9 (64.3)	60 (55.1)	
Overweight/Obese	8 (44.4)	2 (14.3)	22 (34.9)	4 (28.6)	36 (33.0)	
**Mobility** ^1^						<0.001
Low^2^	15 (62.5)	3 (18.8)	17 (23.9)	1 (7.4)	36 (28.8)	
Moderate^3^	6 (25.0)	7 (43.7)	16 (22.5)	2 (14.3)	31 (24.8)	
High^4^	3 (12.5)	6 (37.5)	38 (53.5)	11 (78.6)	58 (46.4)	
**P-ADL Participation** ^**1**^						
Dependent in ≥1 act. of personal care	7 (29.2)	0	6 (8.5)	0	13 (10.0)	0.012
**Balance** ^1^						
Self-rated balance (bad), (n = 114)	16 (69.6)	9 (56.3)	28 (45.2)	6 (46.2)	59 (51.7)	0.232
Fear of falling (yes), (n = 122)	10 (41.7)	5 (31.3)	19 (27.9)	2 (14.3)	36 (29.5)	0.363
**Previous falls**						
**≥**1 fall previous year, (n = 122)	14 (58.3)	6 (40.0)	33 (47.8)	7 (50.0)	60 (49.2)	0.725
**Physical activity**^1^ (n = 118)						0.085
Sedentary	9 (37.5)	1 (6.3)	11 (15.5)	1 (7.4)	22 (17.6)	
Light exercise	13 (54.2)	12 (75.0)	56 (78.9)	12 (85.7)	93 (74.4)	
Hard exercise	0	1 (6.3)	2 (2.8)	0	3 (2.4)	
**Post fracture grip strength (n = 107)**						0.202
Normal (≥10th percentile)	10 (45.5)	4 (30.8)	39 (65.0)	7 (58.3)	60 (56.1)	
Low (5th-10th percentile)	7 (31.8)	3 (23.1)	10 (16.7)	3 (25.0)	23 (21.5)	
Abnormally low (<5th percentile)	5 (22.7)	6 (46.2)	11 (18.3)	2 (16.7)	24 (22.4)	
**Number of chronic diseases** ^**5**^						0.719
0	12 (50.0)	10 (62.5)	35 (49.3)	10 (71.4)	67 (53.6)	
1	7 (29.2)	5 (31.3)	26 (36.6)	3 (21.4)	41 (32.8)	
≥2	5 (20.8)	1 (6.3)	10 (14.1)	1 (7.1)	17 (13.6)	
**Fall-risk-increasing drugs (FRIDs)**						0.946
0 FRIDS	4 (16.7)	4 (25.0)	13 (18.3)	3 (21.4)	24 (19.2)	
Cardiovascular (Cvd) FRIDs	10 (41.7)	4 (25.0)	29 (40.8)	5 (35.7)	48 (38.4)	
Psychotropic (Psy) FRIDs	2 (8.3)	3 (18.8)	6 (8.4)	2 (14.3)	13 (10.4)	
Concomitant Cvd & Psy FRIDs	8 (33.3)	5 (31.3)	23 (32.4)	4 (28.6)	40 (32.0)	

Unit are expressed as number (percentage) unless otherwise stated.

^1^Reported performance of pre-fracture status ^2^Required a walking aid indoors. ^3^Required a walking aid outdoors. ^4^No walking aid required. ^5^According to Charlson’s comorbidity index.

#### Those who fell during positional change had the poorest functional status

Participants whose fall-related fracture occurred during *positional change* appeared to be those with greatest functional limitations. Support for this pattern in the quantitative data is primarily seen in relation to mobility and P-ADL limitations (Table 2). Additionally, this group more frequently, although the difference was not statistically significant, rated themselves as being sedentary, having ‘bad’ balance and having fallen in the year previous to the fracture. In terms of other health characteristics, patterns were somewhat divergent as although these participants reported a high prevalence of comorbidities and FRID use, these differences were not statistically significant from other groups. Nevertheless, support for this pattern was present in the interview data as the majority of those who fell during *positional change* described physiological factors which implicated the presence of chronic conditions or poor health as precipitants for their falls, as was the case in the following example:

*I got up from the chair by the TV and was about to get something for my grandchild…and I’m not even sure what happened, I took a few steps then I just fell, I must have risen too fast and not had the time to get my balance …I didn’t have time to react it happened so fast and my legs are weak.* *(87 year-old female who fell during positional change)*

#### Those who described environmental fall factors (indoors) had moderate physical function but high levels of comorbidity and medication use

The two main described categories of the nature of indoor falls included *physiological* and *environmental*. When compared to those describing falls of *physiological* nature, those describing *environmentally* precipitated falls reported higher levels of physical function (Figure 1; Additional file 1: Table S1). Although not statistically different, these participants less frequently reported their balance as ‘bad’ (p = 0.075) and appeared to have higher levels of mobility (p = 0.461) and functional independence (p = 0.477). Support for stronger beliefs in functional performance was frequently found in interview data where these participants commonly described collisions with furniture or tripping on cords, whilst cleaning indoors, or slipping on surfaces although often moving unhindered in the home, as was the case with this participant:

*I came in from the balcony and my foot slipped somehow in under the edge of the mat, and there is a large plant on that mat so it didn’t follow with me, so I got stuck under it and fell forwards.* *(68 year-old female describing a fall of environmental nature)*

Despite a higher level of self-reported physical function, the group *environmental* had nonetheless a similar prevalence of comorbidity, FRID prescription and previous falls as those perceiving falls as caused by *physiological* factors (Figure 1; Additional file 1: Table S1).

Health characteristics for all indoor fall categories, including *activity-related indoors* and *unknown* are shown in Additional file 1: Table S1.

#### Those who fell outdoors in snow-free environments appeared to have a poorer health than other outdoor groups

Those fracturing outdoors in snow-free environments (*Trips & slips, no snow*) appeared to have lower levels of mobility, a higher incidence of previous falls and showed tendencies towards higher prevalence of disease and use of FRIDs, compared with other outdoor groups (Additional file 1: Table S1). These participants described their falls in terms of environmental factors, often involving unsuitable footwear and/or trips on uneven paths, tree roots or curbsides which were either misjudged or not visible. Not surprisingly, those falling during more *balance challenging* activities and those describing *activity-related* falls reported the least functional limitations and appeared to be those most physically active, both indoors and outdoors.

The health characteristics of those who fractured while standing appeared inconsistent (low levels of observed muscular strength, relatively physically active and relatively low comorbidity levels) and warrant further investigation.

## Discussion

Being descriptive in nature, the present study aimed to identify patterns between fall descriptions and health characteristics of 125 cognitively unimpaired people with hip fracture, who were included from a population-based sample of patients admitted during a 10-month period. Firstly, those who fractured during positional change had the poorest functional status (greatest mobility and P-ADL limitations, poorest self-rated balance, greatest fear of falling and previous falls, and lowest physical activity participation). The majority of these falls were also described in terms of physiological precipitants. Secondly, participants describing indoor falls of environmental nature had a higher reported and observed physical function (fewer mobility and P-ADL limitations, better self-rated balance and hand grip strength) than those describing falls involving physiological factors, but had nonetheless, an equally high prevalence of comorbidities and fall-risk-increasing drug use and previous falls. Thirdly, the health and functional characteristics of those fracturing outdoors in snow-free environments were more similar to those fracturing indoors than to other outdoor groups.

Most previous investigations of fall circumstances have reported falls in general and not specifically those resulting in hip fracture [54-57], which limits the comparability of our findings. We observed however, similar proportions of hip fractures occurring while walking as well as a greater proportion of men who fractured outdoors as previously reported [57].

### Falls during positional change amongst those with poor physical function

Previous studies which have dichotomized falls into indoor or outdoor falls have established that people who fall indoors have poorer health characteristics than those falling outdoors [58-60]. By further division of falls according to activity at the time, our findings go on to suggest that those whose fall occurred during positional change had characteristics implying poorer function than other indoor fallers. Possible explanatory mechanisms for these observed tendencies may be found in the literature. Studies which have examined movement transitions among older people have, for example, demonstrated that those with fear of falling perform sit-to-walk in a way which threatens postural stability [61,62]. Incorrect shifting of bodyweight has also been reported as a major contributor to falls among nursing home dwellers, a population also characterized by poor health status [10]. Practical implications of these findings could involve a focus on task-specific training of muscular strength, postural control and compensatory strategies during chair and bed rises for older people who have fallen during positional change. Additionally, in consideration that many (15/24) of those fracturing during positional change in the current study, described physiological symptoms such as dizziness or disequilibrium, factors such as postural, or orthostatic hypotension may also have precipitated the fall. The diagnosis of orthostatic hypotension, which commonly presents asymptomatically, is not fully defined and it is recognized that good clinical judgment is of importance when investigating and managing this condition [63,64]. Evaluation of orthostatic hypotension may therefore be especially indicated as part of a comprehensive falls assessment among those presenting with falls and fall-related hip fractures following positional change. Further investigation is required in relation to participants who are unable to describe the nature of their falls for two reasons. Firstly, although we observed a tendency for this group to have a poor overall health status, we were unable to draw conclusions due to the small number of these participants (n = 10). Secondly, others report that people who are unable to describe the reason for their fall tend also to restrict their post fall activities and environments [20], which further implies that such individuals are at higher risk for poor outcomes following the fracture.

### Falls of environmental nature amongst those with high levels of comorbidity and medication use

Those who described indoor falls of environmental nature appeared to have higher levels of physical function when compared to those describing falls of physiological nature. Evidence for the association between higher levels of mobility and environmental hazards can be found in the literature [65,66]. However, we also observed equally high levels of comorbidity, FRID use and previous falls among these two groups of indoor fallers. Despite the frequency by which older adults attribute environmental factors for their falls, evidence linking environmental hazards and falls appears weak [28,67,68] and it is sometimes proposed that older people attribute falls to the environment as a strategy to deflect from failing health or feelings of vulnerability [10,69]. Our findings concerning the levels of disease and medication usage among people describing falls of environmental nature may offer further explanation to this pattern. One possible interpretation could be that whilst stronger beliefs in mobility performance predispose these individuals to hazardous or challenging environments, they may, on the other hand, underestimate the adverse effects of comorbidities and medications on their postural control and protective reactions in these situations. Nonetheless, the high prevalence of previous falls among this group highlights the importance that clinicians pose questions concerning fall history and health factors to those presenting with falls, regardless of perceived cause, as medication revision and fall preventive advice may be required. Interestingly, no participant described drug side-effects as a fall precipitant, despite strong evidence in the literature for the association between certain drugs and falls and fractures [42,70,71].

### Health variations according to the nature of outdoor falls

Outdoor falls are generally associated with vigorous elderly people but previous investigations have analysed outdoor falls as a homogenous group [22,58-60]. Our findings suggest that those fracturing outdoors in snow free environments had a poorer health status than other outdoor groups. This implies that not all older people fracturing outdoors can be considered vigorous and those falling under less challenging circumstances may therefore require extra measures to ensure safe mobility and offset future fear of falling or outdoor activity restriction, both commonly arising consequences of hip fracture [72-74]. The role played by the physical environment in falls causation is thought to be mediated by health and function, which in turn increases/decreases an older person’s exposure to environmental hazards or hazardous situations [28,75]. Nevertheless, despite poorer observed general health, no participant who fractured outdoors in snow-free conditions in the current study described their fall in terms of health factors. This further highlights the disparity between older people’s and experts’ accounts of the nature of falls and related fractures. However, due to the small numbers of participants involved in the outdoors groups it is difficult to draw conclusions regarding health patterns and these findings must be regarded as preliminary. Further study is thus required regarding interactions between environmental, health and behavioral factors in relation to hip fracture occurrence and whether perceptions of fall circumstances are associated with the adoption of fall prevention strategies.

### Strengths and limitations

This is the largest study thus far to integrate qualitative data concerning fall descriptions with quantitative data outlining health characteristics and thereby yields a multi-faceted analysis of fall-related hip fractures, which are by nature complex multifactorial events. To ensure data dependability, all interviews were performed by the same investigator who also compared fall accounts with medical records and, when possible, with observer accounts of the fall event. However, falls are often emotive occurrences and it is not possible to fully ensure the accuracy of the fall descriptions. Self-reported functional performance may be influenced by personal and health characteristics and cannot be solely considered a reflection of performance capability. Pre-fracture objective and more detailed measurements of health patterns and functional performance including mobility, physical activity performance, perceived balance, disease severity and timing of medication would have been optimal but were in this study setting unobtainable. We also lacked data concerning bone mineral density, visual capacity and time spent indoors and outdoors. Being a descriptive and explorative study, the statistical analysis is kept simple with cross tabulations of relevant information. To minimize the time elapsed between the fall occurrence and data collection, interviews were performed during hospital stay. This approach may however have negatively affected the measurement of health-related variables such as grip strength. It is also interesting to note that less than one third of all patients with hip fracture, approached during the 10-month study period, were capable of verbally recounting details of their fall. Therefore, our findings cannot be transferred to those with cognitive impairment or others fracturing in institutional care. The health characteristics of our sample correspond, on the other hand, well with community-dwelling patients with hip fracture in the population-based cohort from which they were included [14]. By the descriptive and explorative nature of our study we strived for an inductive approach to the text analysis, not involving testing of a theory or hypothesis. To display relations between study categories, we have therefore limited the statistical analysis to simple cross tabulations [76]. In addition, several of the categories involved in our analysis of patterns were small in number and in many cases the differences observed between the groups were not statistically significant. However, in consideration of the fact that inferential statistics and therefore p-values are dependent on both variance and sample size we are conscious of the fact that that large p-values do not necessarily mean that there is no difference between the groups in our study. Patterns however need to be verified in other populations and our findings may inform and be built upon in further research using qualitative or quantitative methods.

## Conclusions

Our findings indicate that patterns exist in relation to the falls circumstances and health characteristics of people with hip fracture. These patterns, when verified, can provide useful information as to the ways in which fall prevention strategies can be tailored to individuals of varying levels of health and function, who are at risk for falls and hip fracture. Examples of such tailored fall preventive efforts could include: the focus on task-specific training during transfers among frail elderly people, medication review and fall risk education among elderly with a history of falls despite moderate physical function and efforts to enable continued outdoor mobility among less vigorous older people whose falls occur outdoors.

## Additional file

Additional file 1: Table S1. Nature of the fall in relation to health characteristics of 125 interviewed participants. **Legend**: ^1^8/10 occurred indoors. ^2^ Reported performance of pre-fracture status. ^3^ Required a walking aid indoors. ^4^ Required a walking aid outdoors only. ^5^ No walking aid required. ^6^ According to Charlson’s unweighted comorbidity index.

## Abbreviations

ICD-10: International Classification of Diseases, 10th revision
P-ADL: Personal activities of daily living
FRIDS: Fall-risk-increasing drugs
CvdFRIDS: Cardiovascular fall-risk-increasing drugs
PsyFRIDS: Psychotropic fall-risk-increasing drugs
MMSE: Mini-mental State Examination
STS: Sit-to-stand
STW: Sit-to-walk
PA: Physical activity
