# Supplementary material for: The fall descriptions and health characteristics of older adults with hip fracture: a mixed methods study
Source: BMC Geriatr. 2015 Apr 8;15:40. doi: 10.1186/s12877-015-0036-x (PMC4428087; doi:10.1186/s12877-015-0036-x)
Supplement: Additional file 1: Table S1. — Nature of the fall in relation to health characteristics of 125 interviewed participants. Legend: 18/10 occurred indoors. 2 Reported performance of pre-fracture status. 3 Required a walking aid indoors. 4 Required a walking aid outdoors only. 5 No walking aid required. 6 According to Charlson’s unweighted comorbidity index. [file 12877_2015_36_MOESM1_ESM.docx]

**Table 1 (Supporting information)**

|  | **Indoor Falls** | | | **Outdoor falls** | | |  |  |
| --- | --- | --- | --- | --- | --- | --- | --- | --- |
| **Health characteristic, n (%)** | **Environ**  **mental**  **n=32** | **Physio-**  **logical**  **n=35** | **Activity**  **Related**  **n=8** | **Trip/ slips (snow)**  **n=20** | **Trip/ slips (no snow) n=12** | **Activity**  **Related**  **n=8** | **Unknown**  **Nature ^1^**  **n=10** | **Total**  **Falls**  **n=125** |
| **Age group** |  |  |  |  |  |  |  |  |
| 50-80 years | 14 (43.7) | 16 (45.7) | 2 (25.0) | 16 (80.0) | 6 (50.0) | 5 (62.5) | 4 (40.0) | 63 (50.4) |
| 80+ years | 18 (56.3) | 19 (54.3) | 6 (75.0) | 4 (20.0) | 6 (50.0) | 3 (37.5) | 6 (60.0) | 62 (49.6) |
| Gender (female) | 23 (71.8) | 25 (71.4) | 8 (100) | 14 (70.0) | 7 (58.3) | 5 (62.5) | 7 (70.0) | 89 (71.2) |
| **BMI (n=109)** |  |  |  |  |  |  |  |  |
| Underweight | 5 (17.8) | 3 (10.3) | 1 (14.3) | 0 | 0 | 1 (12.5) | 3 (37.5) | 13 (11.9) |
| Normal weight | 14 (20.0) | 15 (51.7) | 5 (71.4) | 14 (73.7) | 6 (60.0) | 5 (62.5) | 1 (12.5) | 60 (55.1) |
| Overweight/Obese | 9 (32.1) | 11 (37.9) | 1 (14.3) | 5 (26.3) | 4 (40.0) | 2 (25.0) | 4 (50.0) | 36 (33.0) |
| **Mobility^2^** |  |  |  |  |  |  |  |  |
| Low ^3^ | 12 (37.5) | 17 (48.6) | 2 (25.0) | 0 | 3 (25.0) | 0 | 2 (20.0) | 36 (28.8) |
| Moderate ^4^ | 9 (28.1) | 8 (22.9) | 2 (25.0) | 5 (25.0) | 1 (8.3) | 2 (25.0) | 4 (40.0) | 31 (24.8) |
| High^5^ | 11 (34.4) | 10 (28.6) | 4 (50.0) | 15(75.0) | 8 (66.7) | 6 (75.0) | 4 (40.0) | 58 (46.4) |
| **P-ADL participation^2^** |  |  |  |  |  |  |  |  |
| Dependent in ≥1 activity of personal care | 3 (9.4) | 6 (17.1) | 0 | 2 (15.4) | 0 | 0 | 2 (20.0) | 13 (10.4) |
| **Balance^2^** |  |  |  |  |  |  |  |  |
| Self-rated balance (bad), (n=114) | 13 (46.4) | 23 (69.7) | 1 (14.3) | 7 (36.8) | 6 (54.5) | 5 (62.5) | 4 (50.0) | 59 (51.7) |
| Fear of falling (yes), n=(122) | 8 (25.0) | 18 (52.9) | 1 (12.5) | 4 (20.0) | 3 (30.0) | 0 | 2 (20.0) | 36 (29.5) |
| **Previous falls** |  |  |  |  |  |  |  |  |
| **≥**1 fall previous year, (n=122) | 18 (56.3) | 18 (51.4) | 3 (37.5) | 6 (30.0) | 7 (58.3) | 2 (25.0) | 6 (60.0) | 60 (48.0) |
| **Physical activity^2^** (n=118) |  |  |  |  |  |  |  |  |
| Sedentary | 7 (22.6) | 9 (28.3) | 0 | 1 (5.3) | 1 (8.3) | 0 | 4 (40.0) | 22 (18.6) |
| Light exercise | 23 (74.2) | 22 (68.7) | 7 (100) | 18 (94.7) | 10 (83.3) | 7 (100) | 6 (60.0) | 93 (78.8) |
| Hard training | 1 (3.2) | 1 (3.1) | 0 | 0 | 1 (8.3) | 0 | 0 | 3 (2.5) |
| **Post fracture grip strength** (n=107) |  |  |  |  |  |  |  |  |
| Normal  (≥10^th^ percentile) | 14 (56.0) | 9 (31.0) | 5 (71.4) | 14(73.7) | 10 (83.3) | 3 (42.8) | 5 (62.5) | 60 (56.1) |
| Low(5^th^-10^th^ percentile) | 5 (20.0) | 9 (31.0) | 2 (28.6) | 1 (5.3) | 2 (16.7) | 2 (28.6) | 2 (25.0) | 23 (21.5) |
| Abnormally low  (<5^th^ percentile) | 6 (24.0) | 11 (37.9) | 0 | 4 (21.1) | 0 | 2 (28.6) | 1 (12.5) | 24 (22.4) |
| **Number of chronic diseases** **^6^** |  |  |  |  |  |  |  |  |
| 0 | 14 (43.7) | 18 (51.4) | 6 (75.0) | 15(75.0) | 6 (50.0) | 3 (37.5) | 5 (50.0) | 67 (53.6) |
| 1 | 9 (28.1) | 1 (31.4) | 2 (25.0) | 5 (25.0) | 5 (41.7) | 4 (50.0) | 5 (50.0) | 41 (32.8) |
| ≥2 | 9 (28.1) | 6 (17.2) | 0 | 0 | 1 (8.3) | 1 (12.5) | 0 | 17 (13.6) |
| **Fall risk increasing drugs (FRIDs)** |  |  |  |  |  |  |  |  |
| 0 FRIDs | 2 (6.3) | 7 (20.0) | 2 (25.0) | 8 (40.0) | 3 (25.0) | 1 (12.5) | 1 (10.0) | 24 (19.2) |
| CvdFRIDs | 15 (46.8) | 12 (34.3) | 1 (12.5) | 7 (35.0) | 3 (25.0) | 6 (75.0) | 4 (40.0) | 48 (38.4) |
| PsyFRIDs | 5 (15.6) | 5 (14.3) | 1 (12.5) | 0 | 1 (8.3) | 1 (12.5) | 0 | 13 (10.4) |
| Concomitant Cvd & PsyFRIDs | 10 (31.3) | 11(31.4) | 4 (50.0) | 5 (25.0) | 5 (41.7) | 0 | 5 (50.0) | 40 (32.0) |
